# BR2 cell penetrating peptide effectively delivers anti-p21Ras scFv to tumor cells with ganglioside expression for therapy of ras-driven tumor

**DOI:** 10.1371/journal.pone.0269084

**Published:** 2022-06-01

**Authors:** Ting Yu, Yingxian Shi, Xinyan Pan, Qiang Feng, Peng Wang, Shuling Song, Lilin Yang, Julun Yang

**Affiliations:** 1 Department of Pathology, 920th Hospital of the Joint Logistics Support Force of PLA, Kunming, Yunnan, People’s Republic of China; 2 The Graduate School, Kunming Medical University, Kunming, Yunnan, People’s Republic of China; 3 Faculty of Medicine, Kunming University of Science and Technology, Kunming, Yunnan, People’s Republic of China; Duke University School of Medicine, UNITED STATES

## Abstract

**Purpose:**

Cell membrane penetrating peptide BR2 can bind with ganglioside and introduce foreign drugs into tumor cells. In this study, we employed BR2 to carry the broad-spectrum anti-p21Ras scFv prepared in our laboratory into ganglioside expressing tumor cells for therapy of ras-driven tumors.

**Methods:**

BR2-p21Ras scFv gene was cloned to prokaryotic expression vector and expressed in *E*. *coli* BL21, then the fusion protein was purified with HisPur Ni-NTA. The immunoreactivity of the fusion protein with p21Ras was detected by ELISA and western blotting. The membrane-penetrating and immune co-localization with p21Ras of the fusion protein were determined by immunofluorescence. The antitumor activity was investigated using MTT, wound healing, colone formation, and apoptosis assays in vitro.

**Results:**

BR2-p21Ras scFv fusion protein was successfully expressed and purified. We found that the fusion protein could specifically penetrate into human tumor cell lines which express ganglioside including human neuroblastoma cell line SK-N-SH, human colon cancer cell line HCT116 and human glioma cell line U251. After entering tumor cells the fusion protein bonded specifically with p21Ras. In vitro experiments revealed that it could significantly inhibit the proliferation, migration, and colone formation of HCT116, SK-N-SH, and U251 cells and promote the apoptosis of these tumor cells.

**Conclusions:**

BR2-p21Ras scFv can penetrate ganglioside expressing tumor cells and inhibit the growth of ras-driven tumor by binding with p21Ras, and producing an inhibitory effect. It is suggested that BR2-p21Ras scFv is a potential ras-driven tumor therapeutic antibody.

## Introduction

It is well known that ras gene is associated with 30% tumorigenesis. p21Ras Protein(p21Ras) is the product of ras gene expression. Previously, we prepared a scFv to p21Ras that could react specifically with wild-type and mutant p21Ras [1]. However, the anti-p21Ras scFv could not penetrate the cell membrane to bind p21Ras within cytoplasm [2,3], therefore it has not been applied to tumor therapy.

BR2 is one of cell membrane penetrating peptides (CPPs) composed of 17 amino acids. The amino acid sequence is RAGLQFPVGRLLRRLLR and the base sequence is CGTGCTGGTTTACAATTTCCTGTTGGCCGCTTCCTTCGACGGCTCCTAAGA.

BR2 is a tumor-specific CPP which can enter cells through lipid mediated phagocytosis by binding to gangliosides (GS) [4]. It suggests that BR2 can be used as a carrier to carry p21Ras scFv into GS expressing cells. GS was highly expressed in most tumor cells and was involved in the process of tumor occurrence, proliferation, invasion, and metastasis [5]. Several studies demonstrated that GS was expressed in almost all types of primary neuroblastoma, with approximately 10^7^ molecules per cell surface [6]. Approximately 75% of primary and metastatic melanomas can detect GS [7]. GS is also highly expressed in other tumors, including osteosarcoma [8], small cell lung cancer [9], liver cancer [10], pancreatic cancer [11], gastric cancer [12], B-cell lymphoma [13], breast cancer [14], and bladder cancer [15].

In the early stage of our study, we used RGD and ACPP to carry the anti-p21Ras scFv to integrin-expressing tumor cells and MMP2-expressing tumor cells, respectively. However, the tumor cells with high GS expression have not been studied. In this study, we constructed BR2-p21Ras scFv (BR2-scFv) fusion protein gene, prepared the fusion protein through prokaryotic expression in E. coli. The fusion protein was obtained with good biological activity after purification and renaturation. The efficiency and antitumor activity of the fusion protein into tumor cells was detected, so as to lay a foundation for clinical application.

## Materials and methods

### Ethical statement

All the study did not involve: Human participants, Human specimens or tissue, Vertebrate animals or cephalopods, Vertebrate embryos or tissues and Field research.

### Cell lines and cell culture

The human neuroblastoma cell line SK-N-SH with N-ras mutation, human colon cancer cell line HCT116 with K-ras mutation, human glioma cell line U251 with wild-type H-p21Ras overexpression, human colonadenocarcinoma celll line CACO-2 without ras mutation but with a few expression of wild-type p21Ras and human normal bronchial epithelial cell line BEAS-2B(without p21Ras expression) were all purchased from the Conservation Genetics CAS Kunming Cell Bank (Kunming, CN). All cells were cultured in medium supplemented containing 10% heat-inactivated foetal bovine serum (FBS) (Biological Industries, Israel), 100 units/mL penicillin, and 100 μg/mL streptomycin under humid conditions at 37°C with 5% CO2.

### Immunohistochemistry

The expression of GS in HCT116 (K-ras mutated cells), SK-N-SH (N-ras mutated cells), U251 (wild-type H-p21Ras overexpression cells), CACO-2 (without overexpression of p21Ras) and normal bronchial epithelial cell line BEAS-2B was detected with immunohistochemistry using an anti-ganglioside antibody (Huabio, ARE6003, China). The cells in logarithmic phase were digested with trypsin, centrifuged, resuspended, and adjusted to a concentration of 2×10^6^/ml to make cell climbing sheets (1x10^5^). The sheets were placed in a culture dish containing 1 ml medium and cultured to logarithmic phase after 10 hours. Next, the cell sheets were fixed with 4% paraformaldehyde and incubated in a wet box at 4°C overnight with the anti-ganglioside antibody (Huabio, ARE6003, China, 1:800), followed by incubation with horseradish peroxidase (HRP)-labelled goat anti-mouse IgG (ZSGB-Bio, ZB-5305, China) and DAB solution. Finally, the expression of ganglioside was estimated by positive staining of the cytoplasmic and plasma membranes.

### Construction of recombinant plasmid and expression bacteria

**Fig 1:**
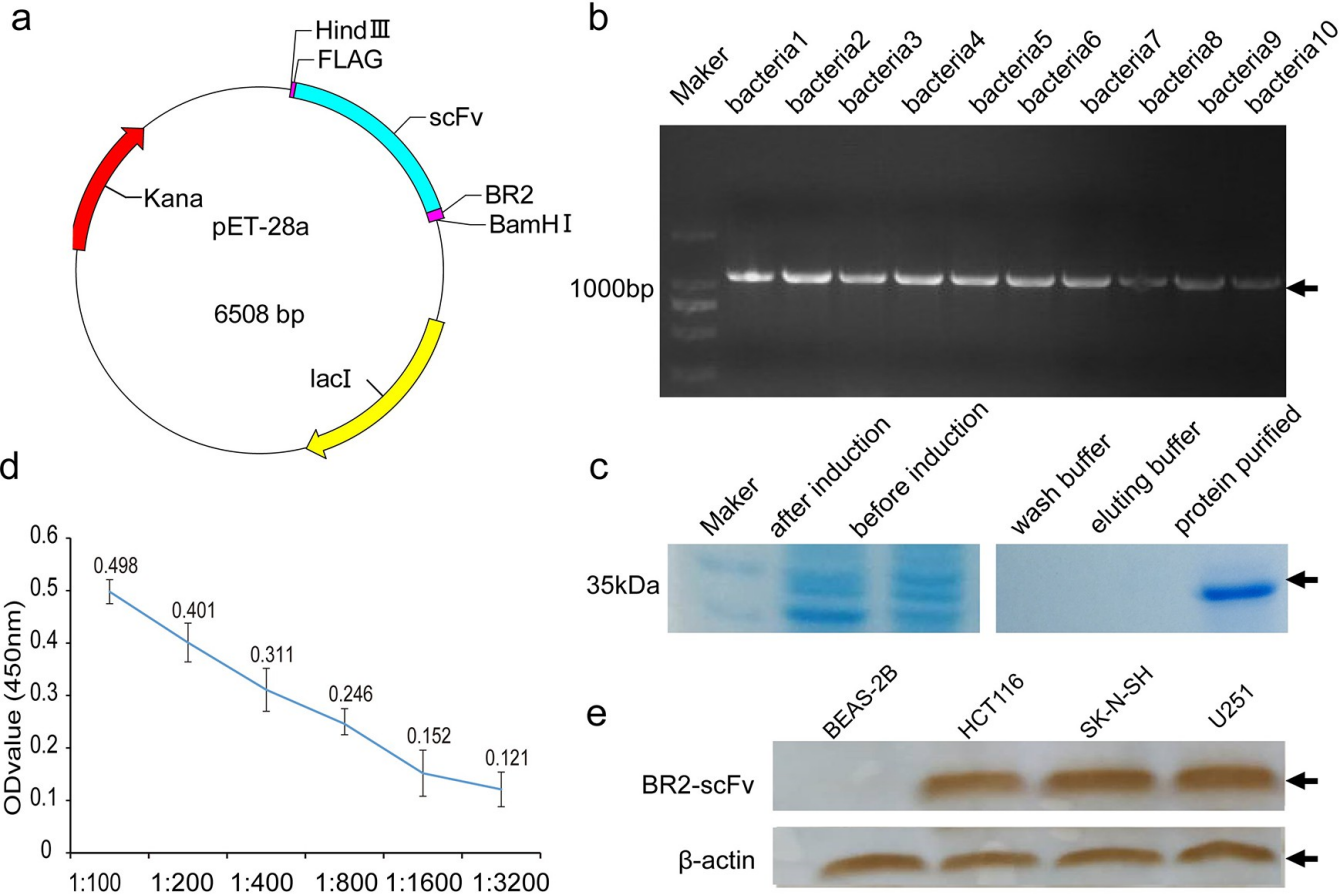
Prokaryotic expression and the immunoreactivity with p21Ras of fusion protein BR2-p21Ras scFv(BR2-scFv). (**a**) Sequences of BR2-scFv were ligated into the pET28a (+) vector to construct recombinant expression plasmids. (**b**) The insertion sequences were confirmed by the PCR results, which showed that the expressing bacterias had thicker gene bands at the target gene bands (1,100 bp) after PCR amplification with the T7 universal primer of the pET-28a (+) plasmid. (**c**) SDS-PAGE analysis showed that the molecular weight of the BR2-scFv was 35 kDa, and the purified fusion protein had no obvious heteroprotein. (**d**) ELISA revealed that the immunizing potency of 0.3 mg/ml BR2-scFv fusion protein was 1:800. (**e**) WB showed that BR2-scFv specifically bind to the p21Ras in tumor cell lines HCT116, SK-N-SH, U251. The target band was not seen in BEAS-2B cells without p21Ras expression.

The BR2-p21Ras scFv gene with the detection tag Flag gene on the back was synthesized(Tsingke Biotechnology Co., Ltd, China). and cloned into the BamHI and HindIII sites of the pET-28a(+) plasmid. The recombinant plasmid contained two His tags for nickel column purification (Fig 1A). Then, the plasmid was transferred into *E*. *coli* BL21(DE3).

### Induced expression, purification and renaturation of the fusion protein

BR2-scFv fusion protein was expressed in *E*. *coli* BL21 after induction with 1 mM IPTG for 5 h at 37°C. Bacteria were harvested by centrifugation at 5,000 g for 15 min at 4°C. Each gram of bacteria was resuspended in 30 ml Tris-HCl buffer (50 mM Tris-HCl, pH 8) and disrupted by sonication (Scientz) at 4°C.

The soluble and insoluble fractions were separated by centrifugation at 13,000 g for 15 min at 4°C. The pellet containing the inclusion bodies was resuspended in wash buffer (20 mM Tris-HCl, 5 mM EDTA and 1% Triton X-100, pH 8.5) at a ratio of 1 g:10 ml and centrifuged at 13,000 g for 15 min at 4°C. The washed inclusion bodies were denatured and solubilized in binding buffer (6 M guanidine hydrochloride, 10 mM imidazole, 0.01 PBS, pH 7.4) for 3 h under static conditions and centrifuged at 14,000 g for 15 min at 4°C. The supernatant was collected for protein purification.

All proteins were affinity-purified using a Hispur Ni-NTA purification kit (NO88229, Thermo, Germany). In brief, the Ni-NTA His-Bind Resin was packed into a column equilibrated with binding buffer. The binding buffer was slowly applied to the column and centrifuged at 700 g for 2 min at 4°C. This step was repeated until all the binding buffer passed through the column, after which wash buffer (6 M Guanidine hydrochloride, 25 mM imidazole, 0.01 MPBS, pH 7.4) was applied. When the concentration of washed protein was detected by A280 spectrophotometer until it was less than 0.01, the target proteins could be eluted. The BR2-scFv fusion protein was eluted with elution buffer (6 M guanidine hydrochloride, 250 mM imidazole, 0.01 MPBS, pH 7.4) and analysed with dodecyl sulfate, sodium salt-Polyacrylamide gel electrophoresis(SDS-PAGE).

The BR2-scFv fusion protein was refolded by dialysis in dialysis refolding solution containing 200 mM NaCl, 20 mM Tris-HCl, and 2 mM GSH, pH 8, with decreasing concentrations of hydrochloride (4 M, 2 M, 1 M, and 0 M). Each dialysis step was performed at 16°C for 12 h with a magnetic stirrer. Then, the BR2-scFv fusion protein was dialyzed in 0.01 M PBS (pH 8.0) containing 2 M NaCl for 3 hours and stored at -20°C after recovery.

### Enzyme Linked Immunosorbent Assay (ELISA)

The binding specificity of the antibody BR2-scFv fusion protein to antigen N-p21Ras was determined by ELISA. Ninety-six-well EIA/RIA plates (Corning) were coated for 12 h at 4°C with 5 μg/ml antigen N-p21Ras, and then blocked with 1% BSA-PBS blocking solution for 1 h at 37°C. After washing with PBST (0.02%KH_2_PO_4_, 0.29%Na_2_HPO_4_-12H_2_O, 0.8%NaCl, 0.02%KCL, 0.05%Tween-20), 0.3 mg/ml BR2-scFv was diluted with 0.1%BSA in different gradients (1:100, 1:200, 1:400, 1:800, 1:1600, and 1:3200) and applied to each well for 1 h at 37°C. After washing with PBST, bound protein was detected by the anti-FLAG tag antibody (Abnova, MAB9744, China) and horseradish peroxidase (HRP)-labelled goat anti-mouse IgG (ZSGB-Bio, ZB-5305, China). Each well was then stained with TMB (3, 30, 5, and 50-tetramethylbenzidine). After colour development for 10 minutes, the termination solution was added. Absorbance was read at 450 nm wavelength on the enzyme-linked immunosorbent assay (Bio-Rad, Model 680, USA).

### Western blot assay(WB)

WB assay was performed by extracting protein from cell samples with RIPA cellular lysate (Solarbio), followed by electrophoresis on SDS-PAGE gels and transfer of proteins to polyvinylidene fluoride (PVDF) membranes. Next, the PVDF membranes were incubated with BR2-p21Ras scFv (0.3mg/ml,dilution1:800) after using skimmed milk powder as a blocking agent and then were incubated with the anti-FLAG tag antibody(Abnova,MAB9744,China) at a 1:1000 dilution and horseradish peroxidase (HRP) labeled goat anti-mouse IgG (ZSGB-Bio, ZB-5305, China) at 1:1000. After washing with TBST (50mMTris, 150mMNaCl, and 0.5%Tween-20)for three times, The PVDF membranes were then stained with DAB. β-Actin (ZSGB-Bio,TA-09,China) protein was used as an internal control.

### Immunofluorescence assay

When the cell climbing sheets grew to the logarithmic phase for 24 h, 2 μM BR2-scFv, BR2, and PBS was added, respectively and cocultured for 12 hours. The cells slides were fixed (4% paraformaldehyde for 30 minutes), permeabilized (0.2% Triton 100 for 20 minutes), and then internalized BR2-scFv were detected with the anti-FLAG tag antibody (Abnova, MAB9744, China) for 1 h at 37°C. Subsequently, rhodamine-labelled goat anti-rabbit IgG antibody (1:150) was incubated for 1 h at 37°C in the dark. The nuclei were stained with DAPI, and the results were observed under a fluorescence microscope.

### Immune co-localization assay

The cell lines were seeded on coverslips and cultured in dishes at 37°C with 5% CO2, when 80% confluent cells were formed. BR2-scFv was added, and cocultured for 7 hours. And then fixed with 4% paraformaldehyde for 30 min and permeabilized with 0.2% Triton 100 for 20 minutes.The slides were incubated overnight at 4°C with primary rabbit anti-His Tag mAb (clone number: D3I1O, Cell Signaling TECHNOLOGY, USA) and mouse pan-Ras mAb (clone number: C4, SANTA CRUZ, USA), washed for 5 min with PBS then incubated for 1h at 37°C in the dark with FITC-conjugated goat anti-rabbit antibody (ZSGB-BIO) and TRITC-conjugated goat anti-mouse antibody (ZSGB-BIO). The nuclei were stained with DAPI, and the results were observed under a fluorescence microscope.

### Cell Proliferation Assays (MTT Assay)

To assess the killing effects of BR2 and BR2-scFv fusion protein, HCT116, SK-N-SH, U251, BEAS-2B, and CACO-2 cells were plated at a density of 2x10^4^ cells per well in 96-well plates. After 24 h of incubation, cells were treated with BR2 or BR2-scFv fusion protein (0, 1, 2, and 5 μM). Then, 20 μl of (4,5-dimethylthiazol-2-yl)-2,5-diphenyltetrazolium bromide (MTT) (5 mg/ml, pH = 7.4; Amresco, USA) was added to the cultured cells in each well according to the manufacturer’s instructions (Amresco, USA). Then, DMSO (100 μl per well) was added and the plates were shaken for 10 min. The optical density value of each well was read at 490 nm using a microplate reader (Bio-Rad, Model 680, USA). Cell viability was expressed as the percentage of viable cells treated with the BR2 or BR2-scFv fusion protein compared to the PBS-treated control (100%). All experiments were performed in triplicate.

### Cell migration assay

HCT116, SK-N-SH, U251 BEAS-2B, and CACO-2 cells in the logarithmic growth phase were collected and seeded in a 6-well plate (1 x 10^6^ cells per well), and incubated at 37°C in DMEM or RPMI 1640 medium supplemented with 10% FBS until the cells reached 95% confluence. Micropipette tips (20 ml) were used to make vertical scratches in the 6-well plate. PBS was used to remove the falling cells, 2 μM BR2 or BR2-scFv was added to the experimental groups, and an equal volume of PBS was added to the PBS group. At 0, 24, and 48 h after scratching, 3 fields were selected in each group and photographed under an inverted microscope (Olympus, Japan). ImageJ software was used to calculate the scratch area and cell migration. Cell migration (%) = (scratch area before 24 h or 48 h—scratch area after 24 h or 48 h)/scratch area before 24 h x 100%.

### Colony formation analysis

Approximately 1 x 10^3^ cells in 2 ml of DMEM or RPMI 1640 containing 10% FBS were cultured in 6-well plates at 37°C with 5% CO2 and treated with 2 μM BR2 or BR2-scFv in growth medium every 72 h for 14-21 days. Following treatment, the cell colonies were fixed with methanol, stained with Giemsa (Solarbio, USA, G8220), photographed, and analysed with ImageJ software. Colonies with a diameter >200 mm were counted, and the clone formation rate was calculated. Clone formation rate (%) = (clone number/inoculated cell number) x100%.

### TUNEL assays

HCT116, SK-N-SH, U251 BEAS-2B, and CACO-2 cell climbing sheets (1x10^5^) were treated with 2 μM BR2, BR2-scFv, or PBS for 24 h and then fixed with 4% paraformaldehyde. Apoptosis was detected by TUNEL assay according to the protocol (In Situ Cell Death Detection Kit; Roche Diagnostics). The apoptosis of tumor cells was visualized using a fluorescence microscope (Olympus, Japan), and the percentages of positive cells in five randomly selected 100x objective fields (3.8 mm^2^) were calculated.

### Statistical analysis

Statistical analyses were performed using SPSS Version 22.0. Data were expressed as the mean value ± s.d. Statistical significance was determined with one-way ANOVA and the Student-Newman-Keuls method. Statistical significance was indicated by a value of P<0.05.

## Results

### Expression and purification of BR2-scFv fusion protein

The construction of prokaryotic recombinant expression plasmid which carried BR2-scFv gene was shown in Fig 1A. The recombinant plasmid was coloned to E. coli BL21 bacteria successfully. The insertion sequences were detected in expressing bacteria by polymerase chain reaction (PCR) analysis (Fig 1B). The fusion protein with MW of 35 kDa were expressed in *E*. *coli* BL21 under the best inducing conditions of 1 mM IPTG for 5 h at 37°C (Fig 1C). Then, the samples were purified using glutathione agarose affinity resin with a nickel column. The purified proteins were resolved by SDS-PAGE electrophoresis at 35 kDa (Fig 1C). After protein renaturation, ELISA showed that the protein we obtained had immunoactivity and was able to bind to the p21Ras. The immunizing potency of 0.3 mg/ml BR2-scFv fusion protein was 1:800 (Fig 1D). WB further confirmed that BR2-scFv fusion protein specifically bound to the p21Ras in the HCT116, SK-N-SH and U251 cells (Fig 1E).

### BR2-scFv fusion protein penetrate living cells and bind to the p21Ras in cells

**Fig 2:**
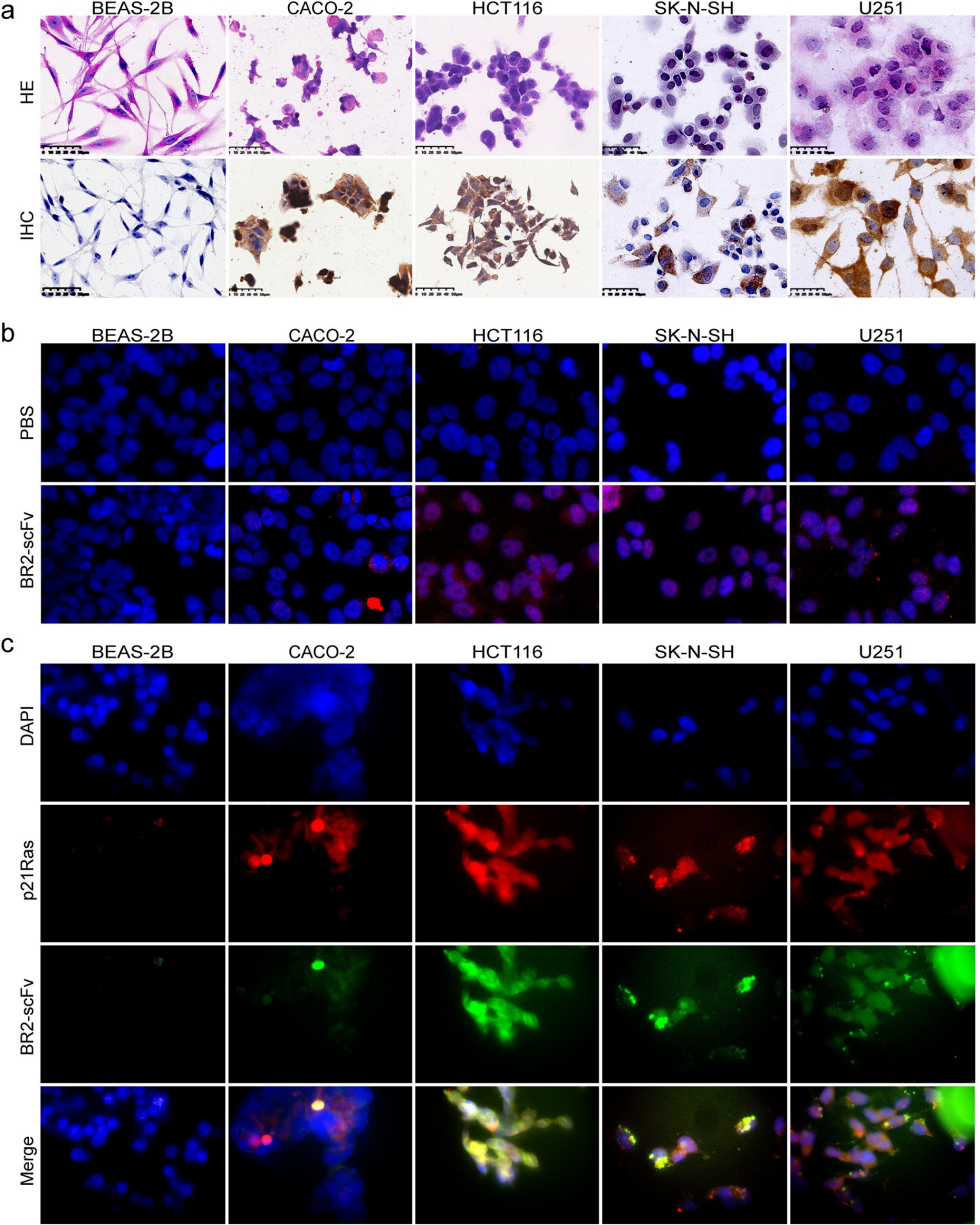
BR2-p21Ras scFv(BR2-scFv) could enter GS expressing tumor cells and bind to p21Ras. (**a**) HE staining of cell climbing sheets showed that normal cells BEAS-2B were long spindle shaped and had benign characteristics, including single existence, consistent size and shape, oval nucleus and no atypia. Tumor cells HCT116, SK-N-SH, U251 and CACO-2 have obvious atypia, including mass growth, different size and morphology, increased nuclear to cytoplasmic ratio, large and deep staining of nuclei, and irregular nuclear morphology.Immunohistochemistry (IHC) demonstrated that that normal cell BEAS-2B did not express Ganglioside(GS), and tumor cells HCT116, SK-N-SH, U251 and CACO-2 expressed GS in cytoplasm and membrane (yellow). (**b**) After co-culture with BR2-scFv, BR2-scFv was detected by immunofluorescence microscopy in HCT116, SK-N-SH, U251 and CACO-2 cells, but none was detected in BEAS-2B cells. Red: BR2-scFv, Blue: Nuclei. (**c**) The co-localization of BR2-scFv and p21Ras in HCT116, SK-N-SH, U251 and CACO-2 cells was observed by immunofluorescence microscopy. But no co-localization staining was found in normal cell BEAS-2B. Red: p21Ras, green: BR2-scFv, yellow: Merged image of the two fluorescence patterns. Nuclei were colourationed with DAPI (blue).

The IHC assay showed that the expression of GS in HCT116 cells, SK-N-SH cells, U251 cells and CACO-2 cells but not in BEAS-2B cells (Fig 2A). We next evaluated whether BR2-scFv fusion protein can reach the cytosol after coculture by immunofluorescence microscopy. We found that there were strong red immunofluorescence of BR2-scFv in HCT116, SK-N-SH and U251 cells, weak red immunofluorescence in CACO-2 cells but no BR2-scFv immunofluorescence in BEAS-2B cells (Fig 2B). Then, double color immunofluorescence staining demonstrated that BR2-scFv and the p21Ras were significantly bound together in HCT116, SK-N-SH, U251 and CACO-2 cells which expressed GS (Fig 2C). These results demonstrate that BR2-scFv fusion protein can specifically recognize the GS of tumor cells and bind to the p21Ras at the inner plasma membrane after cellular internalization and cytosolic localization.

### BR2-scFv fusion protein inhibit growth of tumor cells in vitro

**Fig 3:**
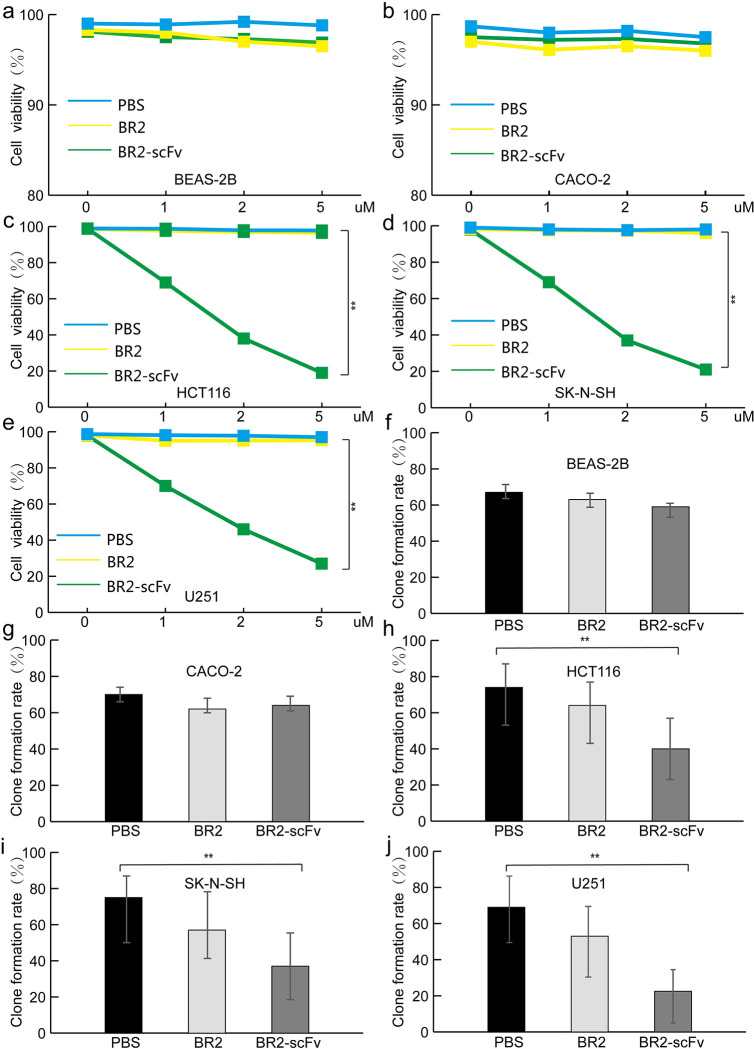
Effect of BR2-p21Ras scFv(BR2-scFv) on cell viability and clone formationm. (**a**-**e**) HCT116, SK-N-SH, U251, CACO-2 and BEAS-2B cells were treated with PBS, BR2, or BR2-scFv (0, 1, 2, and 5 μM) and incubated at 37°C for 24 h. Cell viability was measured with the MTT assay. Cell viability of tumor cells in the BR2-scFv group was significantly lower than that in the BR2 and PBS groups in HCT116, SK-N-SH, and U251 cells. (**f**-**j**) A clone formation experiment was performed to detect the effect of BR2-scFv on tumor cells proliferation. Tumor cells were incubated with 2 μM fusion protein. After 2 weeks of incubation, monoclonal cells were stained with Giemsa. The clone formation rate of tumor cells in the BR2-scFv group was also significantly lower than that in the BR2 and PBS groups in HCT116, SK-N-SH, and U251 cells.

The effect of BR2-scFv fusion protein on the biological behaviour of tumor cells was assessed under monolayer culture conditions. MTT assays were performed to evaluate the potential killing ability of BR2-scFv fusion protein in HCT116, SK-N-SH, and U251 cells. In contrast to BR2-infected group and PBS group, the viability of 5 μM BR2-scFv fusion protein-treated group was 20.33±5.13%, 21.26±7.38%, and 23.31±6.46% in HCT116, SK-N-SH, and U251 cells. This was much lower than that of BR2-infected or PBS-treated cells, which showed negligible cytotoxicity to cells (Fig 3A–3E). The BR2-scFv fusion protein-infected group exhibited dose-dependent antiproliferative activity against ras-driven tumor cells. There was a significant difference among the data of the three groups (P<0.01), indicating that BR2-scFv fusion protein could significantly inhibit the growth and proliferation of tumor cells.

The clone formation test was performed to evaluate the proliferation effects of BR2-scFv fusion protein in HCT116, SK-N-SH, and U251 cells. The BR2-scFv fusion protein-infected group clone formation rates were 40.38±6.20%, 33.6±5.48%, and 22.67±1.52% in HCT116, SK-N-SH, and U251 cells, respectively. The BR2-infected group clone formation rates were 65.3±7.62%, 58.93±4.34%, and 54.56±8.41% in these cells (Fig 3F–3J). The results of the cell clone formation test showed that the BR2-scFv fusion protein group could significantly inhibit the proliferation of HCT116, SK-N-SH, and U251 cells.

**Fig 4:**
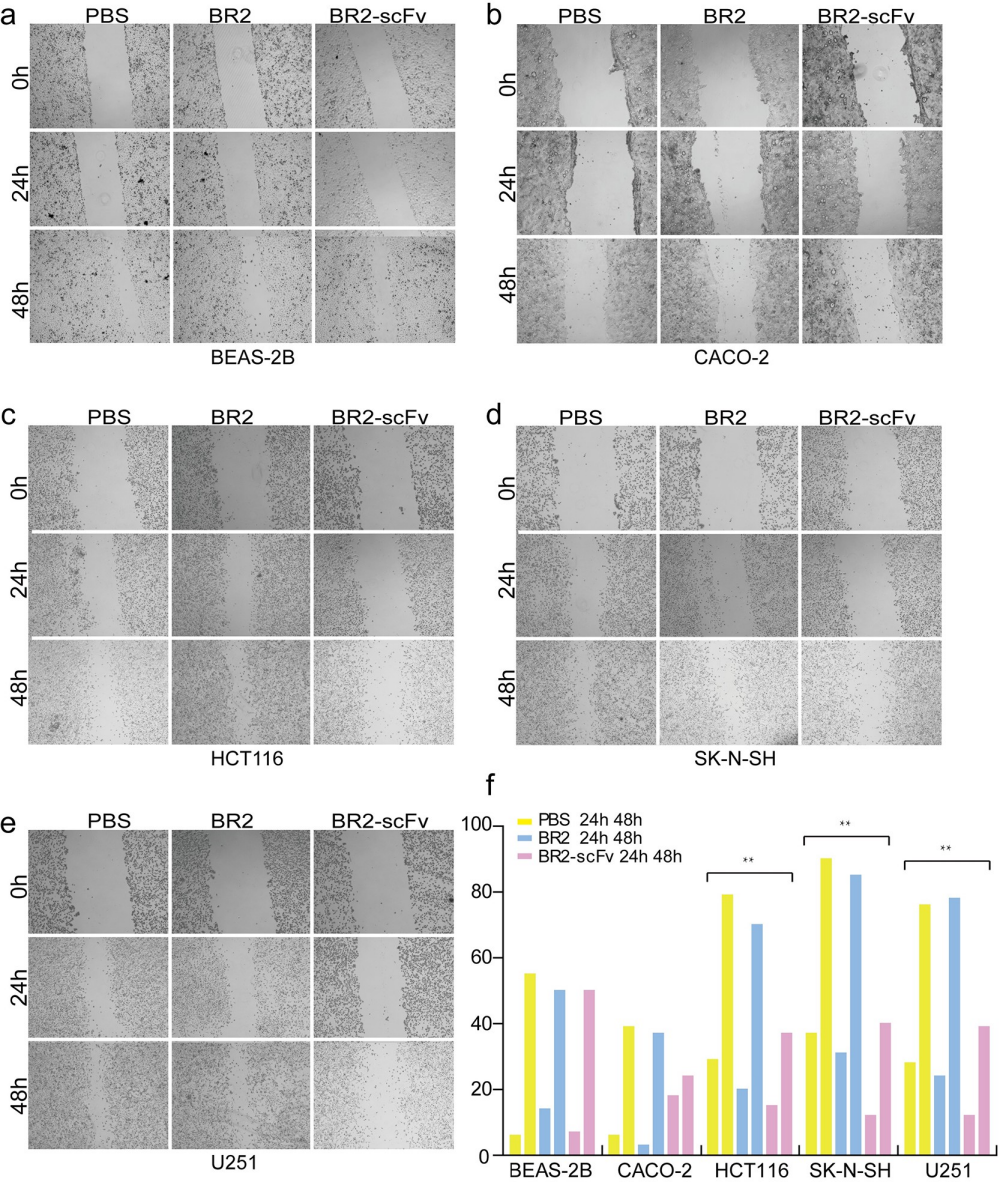
The antitumor efficacy of BR2-p21Ras scFv(BR2- scFv) in vitro. (**a**) The normal cell BEAS-2B without GS was no significant difference of wound healing ability among PBS、BR2 and BR2- scFv treatment group. (**b**) Human colonadenocarcinoma celll line CACO-2, which expressed GS but did not have ras mutation, was no obvious distinctions of wound healing ability in BR2- scFv group compared with the control group PBS and BR2 (**c-e**). The wound healing ability of the BR2- scFv group was slower than the BR2 and PBS groups at 24 and 48 h in HCT116(c), SK-N-SH(d), and U251 cells(e). (**f)** Calculating the percentage of migrating cells, the BR2-scFv group was lower than the BR2 and PBS groups in HCT116, SK-N-SH, and U251 cells at 24 and 48 hours.

A scratch test was performed on HCT116, SK-N-SH, and U251 cells that were infected with BR2-scFv fusion protein for 24 h and 48 h, and the healing of the scratches was observed. The percentages of HCT116, SK-N-SH, and U251 cell scratches were 15.75±2.09%, 16.48±2.68%, and 16.65±2.18% at 24 h, and 39.65±5.27%, 43.57±4.98%, and 39.38±3.77% at 48 h after infected with BR2-scFv. The percentages of these cell scratches were 25.59±4.42%, 34.3±4.45%, and 28.14±3.20% at 24 h, and 73.8±4.78%, 85.7±5.373%, and 74.03±2.05% at 48 h in the control group (BR2-infected) (P<0.05) (Fig 4). These results suggested that BR2-scFv fusion protein could effectively inhibit the migration ability of HCT116, SK-N-SH, and U251 cells.

**Fig 5:**
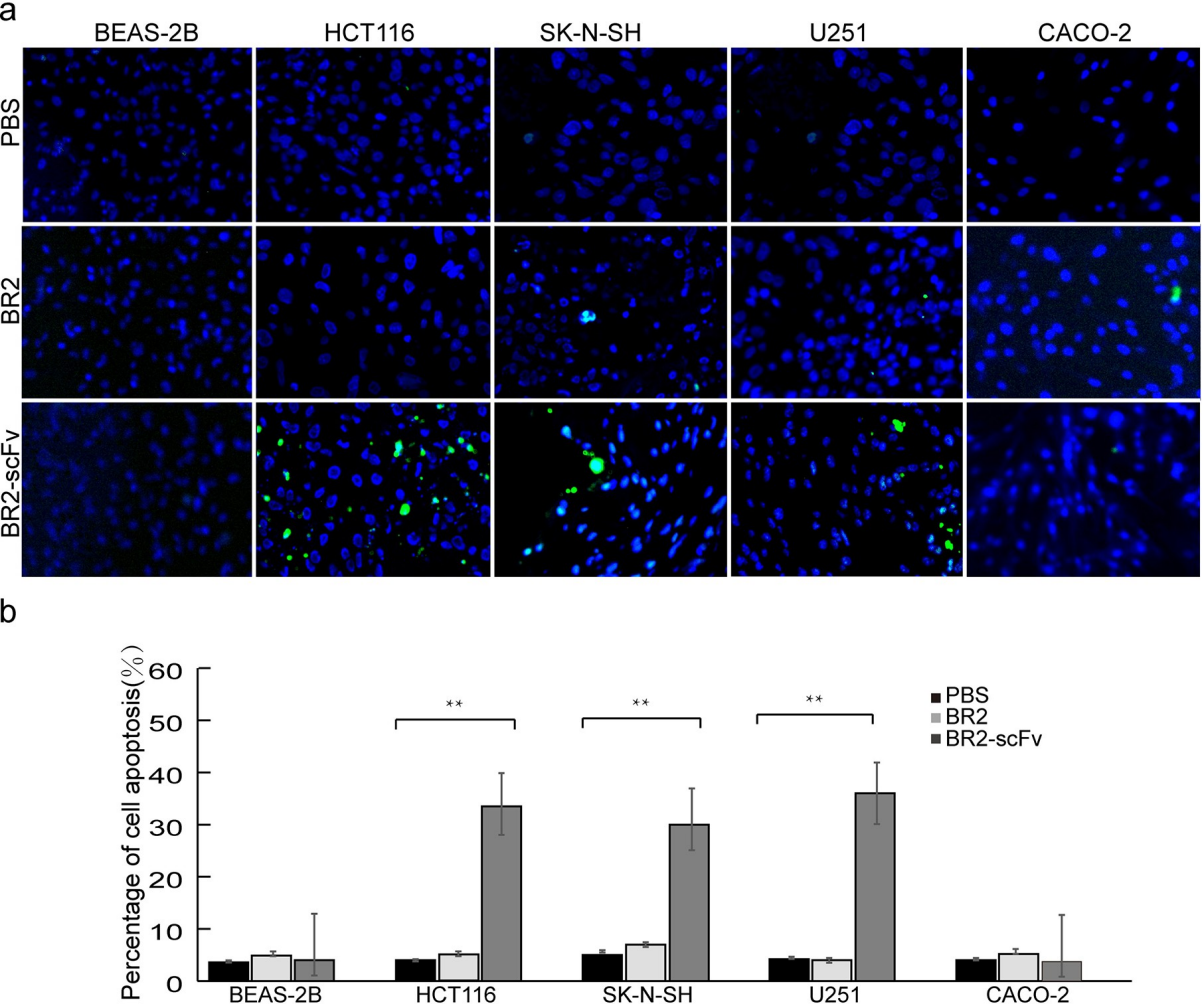
The apoptosis of tumor cells was detected by TUNEL. (**a**) In the normal cell BEAS-2B, almost no apoptotic cell was found in the BR2-scFv treatment group. In HCT116, SK-N-SH and U251 tumor cells the number of apoptotic cells (green fluorescence) in the BR2-scFv group was more than that in the BR2 and PBS groups. (**b**) The percentage of these apoptotic tumor cells in the BR2-scFv group was higher than that in the BR2 and PBS groups.

The apoptosis of HCT116, SK-N-SH, and U251 cells was examined using the TUNEL assay. The number of apoptotic HCT116, SK-N-SH, and U251 cells increased obviously after BR2-scFv fusion protein infection, and there were almost no apoptotic cells in the BR2-fected cell group. The percentage of apoptosis was significantly higher in the BR2-scFv fusion protein infected group (33.28±3.47%, 30.86±8.81%, and 35.71±5.67%) than in the BR2-fected control group (5.40±0.64%, 6.21±5.38%, and 3.84±0.25%) (P<0.01) (Fig 5). The TUNEL results indicated that BR2-scFv fusion protein could significantly promote the apoptosis of HCT116, SK-N-SH, and U251 cells.

## Discussion

Currently, CPPs, as a worthy short peptide, are able to deliver various macromolecules into cells. Especially in cancer research, CPPs are a significant therapeutic delivery tool [16–18]. By CCPs delivery, proteins, plasmids, siRNA, nanoparticles, PNAs, and liposomes, which are not able to enter cells alone have been transported into the cells [4,19–21]. CPPs are classified into three categories according to their physicochemical properties: cationic, amphiphilic, and hydrophobic. Most CPPs are cationic because of their positive charge [18,22]. Tumor-specific CPPs penetrate tumor cells by binding with their receptors on the cell surface [23–25]. The integrin is the receptor of RGD cell membrane penetrating peptide, Huang et al in our laboratory used RGD to delivery anti-p21Ras scFv into integrin expressing tumors [26,27]. The matrix metalloproteinase 2(MMP2) is the receptor of activatable cell-penetrating peptide ACPP, Du et al in our laboratory used ACPP cell membrane penetrating peptide to delivery anti-p21Ras scFv into MMP2 expressing tumors [28,29]. The research results mentioned above show that both RGD and ACPP can successfully carry scFv into specific tumor cells, and then inhibit tumor growth. GS is the receptor for BR2,it can successfully carried liposomes containing anticancer drug cantharidin into liver cancer cells and then significantly inhibited the growth of transplanted tumor [30].

Here, we employed BR2 to help anti-p21Ras scFv into GS expressing tumor cells. Through immunofluorescence experiment, we found that BR2-p21Ras scFv fusion protein was efficiently internalized into K-ras mutated colon cancer HCT116 cells, N-ras mutated neuroblastoma SK-N-SH cells, and human glioma cell line U251 with wild-type H-p21Ras overexpression. Immune co-localization test showed that BR2-p21Ras scFv could significantly bind to mutated ras or wild-type overexpressed p21Ras in HCT116, SK-N-SH and U251 cells. The observation effect of confocal microscope is obviously better than that of fluorescence microscope, but there was no confocal microscope at that time, which is a defect of this paper. All these findings suggest that after the BR2-p21as scFv fusion protein penetrates the tumor cell membrane, they could specifically bind to the target p21Ras. Other studies have also proved that BR2 effectively transports therapeutic proteins or anticancer drugs to target cells [31].

We further studied the effect of BR2-p21Ras scFv on the biological behaviour of tumor cells in vitro. MTT, clone formation, wound healing and TUNEL experiments revealed that the fusion protein can inhibit tumor cells growth and promote tumor cells apoptosis.

Compared with the anti-p21Ras scFv carried by RGD or ACPP, the anti-p21Ras scFv carried by BR2 into tumor cells had the same antitumor effect [26,29]. The receptor of RGD is integrin, the receptor of ACPP is MMP2, and the receptor of BR2 is GS. The anti-p21Ras scFv carried by BR2 can effectively treat tumor cells with high GS expression. Therefore, the therapeutic range of ras-driven tumors is expanded.

In our previous studies, we found that intracellular anti-p21Ras scFv could inhibit the expression of human liver cancer cell line BEL-7402 ras downstream genes including MAPK1, PI3K and PLCε [3]. So the expression of ras downstream genes were not detected here.

Moreover, The fusion protein was prepared by prokaryotic expression system, which can be used for large-scale shake flask fermentation under laboratory conditions. It is reported that prokaryotic expression system has expressed fusion proteins containing BR2 and scFv genes [4]. After HisPur Ni-NTA purification, the relatively pure fusion protein was obtained. 16.4mg fusion protein was obtained after purification of 1.5L bacterial solution.

Taken together, We successfully used BR2 as the carrier of anti-p21Ras scFv to help it enter tumor cells and exert antitumor activity. The BR2-p21Ras scFv fusion protein may be extensively used for ras-driven tumor therapy in the future.

## Supporting information

S1 Fig Fig 1F original underlying images. (TIF)

S2 Fig Fig 1B and 1C original underlying images. (PNG)

## References

[1] Yang JL, Liu DX, Zhen SJ, Zhou YG, Zhang DJ, Yang LY, et al: A novel anti-p21Ras scFv antibody reacting specifically with human tumour cell lines and primary tumour tissues. *BMC Cancer* 2016, 16:131. doi: 10.1186/s12885-016-2168-6 26897358 PMC4761205

[2] Pan XY, Liu XJ, Li J, Zhen SJ, Liu DX, Feng Q, et al: The antitumor efficacy of anti-p21Ras scFv mediated by the dual-promoter-regulated recombinant adenovirus KGHV300. *Gene Ther* 2017, 24(1):40–48. doi: 10.1038/gt.2016.74 27834948

[3] Yang JL, Pan XY, Zhao WX, Hu QC, Ding F, Feng Q, et al: The antitumor efficacy of a novel adenovirus-mediated anti-p21Ras single chain fragment variable antibody on human cancers in vitro and in vivo. *Int J Oncol* 2016, 48(3):1218–1228. doi: 10.3892/ijo.2016.3334 26780944

[4] Lim Ki Jung, Sung Bong Hyun, Shin Ju Ri, Lee Young Woong, Kim Da Jung, Yang Kyung Seok, et al: A Cancer Specific Cell-Penetrating Peptide, BR2, for the Efficient Delivery of an scFv into Cancer Cells. *PLoS ONE* 2013, 8(6):e66084. doi: 10.1371/journal.pone.0066084 23776609 PMC3679022

[5] Ning N, Chen NH: [Progress in the research of ganglioside’s biological activities]. *Sheng Li Ke Xue Jin Zhan* 2009, 40(1):24–30. 19408699

[6] Schulz G, Cheresh DA, Varki NM, Yu A, Staffileno LK, Reisfeld RA: Detection of Ganglioside G D2 in Tumor Tissues and Sera of Neuroblastoma Patients Detection of Ganglioside GD2in Tumor Tissues and Sera of Neuroblastoma. *Cancer Res* 1984, 44(12):5914–5920. 6498849

[7] Tsuchida T, Saxton RE, Irie RF: Gangliosides of human melanoma: GM2 and tumorigenicity. *J Natl Cancer Inst* 1987, 78(1):55–60. doi: 10.1093/jnci/78.1.55 3467130

[8] Heiner JP, Miraldi F, Kallick S, Makley J, Neely J, Smith-Mensah WH, et al: Localization of GD2-specific monoclonal antibody 3F8 in human osteosarcoma. *Cancer Res* 1987, 47(20):5377–5381. 3115567

[9] Zheng C, Terreni M, Sollogoub M, Zhang Y: Ganglioside GM3 and Its Role in Cancer. *Current Medicinal Chemistry* 2018, 25(16):2933–2947. 10.2174/0929867325666180129100619 29376491

[10] Qamsari ES, Nourazarian A, Bagheri S, Motallebnezhad M: Ganglioside as a Therapy Target in Various Types of Cancer. *Asian Pacific Journal of Cancer Prevention Apjcp* 2016, 17(4):1643. doi: 10.7314/apjcp.2016.17.4.1643 27221833

[11] Chu KU, Ravindranath MH, Gonzales A, Nishimoto K, Tam WY, Soh D, et al: Gangliosides as targets for immunotherapy for pancreatic adenocarcinoma. *Cancer* 2015, 88(8):1828–1836. 10760759

[12] Dohi T, Ohta S, Hanai N, Yamaguchi K, Oshima M: Sialylpentaosylceramide detected with anti-GM2 monoclonal antibody. Structural characterization and complementary expression with GM2 in gastric cancer and normal gastric mucosa. *J Biol Chem* 1990, 265(14):7880–7885. 2139874

[13] Anh-Tuan N, Pick J, Mod A, Hollan SR: Gangliosides in acute myeloid leukaemia (AML) and non-Hodgkin’s lymphoma (NHL). *Eur J Cancer Clin Oncol* 1986, 22(8):1003–1007. doi: 10.1016/0277-5379(86)90068-4 3464433

[14] Bitton R, Guthmann M, Gabri M, Carnero A, Alonso D, Fainboim L, et al: Cancer vaccines: An update with special focus on ganglioside antigens (Review). *Oncology Reports* 2002, 9(2):267–276. 11836591

[15] Venkatrao V, Ramya DS, Ambati CR, Jin F, Vasanta P, Khoa N, et al: Expression of ganglioside GD2, reprogram the lipid metabolism and EMT phenotype in bladder cancer. *Oncotarget* 2017, 8(56):95620–95631. doi: 10.18632/oncotarget.21038 29221154 PMC5707048

[16] Matijass M, Neundorf I: Cell-penetrating peptides as part of therapeutics used in cancer research. *Medicine in Drug Discovery* 2021, 23(2):295–322.

[17] Bohmova Machova, Pechar Pola, Venclikova Janouskova, et al: Cell-Penetrating Peptides: a Useful Tool for the Delivery of Various Cargoes Into Cells. *Physiological research / Academia Scientiarum Bohemoslovaca* 2018, 67(2):S267–S279. doi: 10.33549/physiolres.933975 30379549

[18] Sajid MI, Moazzam M, Stueber R, Park SE, Cho Y, Malik NUA, et al: Applications of amphipathic and cationic cyclic cell-penetrating peptides: Significant therapeutic delivery tool. *Peptides* 2021, 141:170542. doi: 10.1016/j.peptides.2021.170542 33794283

[19] Falato L, Gestin M, Ü. L: Cell-Penetrating Peptides Delivering siRNAs: An Overview. *Methods Mol Biol* 2021, 2282(1):329–352. doi: 10.1007/978-1-0716-1298-9_18 33928583

[20] Zhu P, Jin L: Cell Penetrating Peptides: A Promising Tool for the Cellular Uptake of Macromolecular Drugs. *Curr Protein Pept Sci* 2018, 19(2):211–220. doi: 10.2174/1389203718666170710115240 28699510

[21] Mae M, Langel U: Cell-penetrating peptides as vectors for peptide, protein and oligonucleotide delivery. *Curr Opin Pharmacol* 2006, 6(5):509–514. doi: 10.1016/j.coph.2006.04.004 16860608

[22] Guo Z, Peng H, Kang J, D. S: Cell-penetrating peptides: Possible transduction mechanisms and therapeutic applications. *Biomed Rep* 2016, 4(5):528–534. doi: 10.3892/br.2016.639 27123243 PMC4840506

[23] K. MR, Sushmita S, G. RS, Vishal S, J. BA, S. LT, et al: Disruptin, a cell-penetrating peptide degrader of EGFR: Cell-Penetrating Peptide in Cancer Therapy *Translational Oncology* 2021, 14(8):101140. doi: 10.1016/j.tranon.2021.101140 34107419 PMC8187233

[24] Hamilton AM, Aidoudi-Ahmed S, Sharma S, Kotamraju VR, Foster PJ, Sugahara KN, et al: Nanoparticles coated with the tumor-penetrating peptide iRGD reduce experimental breast cancer metastasis in the brain. *J Mol* Med (Berl) 2015, 93(9):991–1001. 25869026 10.1007/s00109-015-1279-x PMC4807972

[25] Anja G, Mareike H, Ivan R, József T, Sergio MV, Klaus S, et al: Characterization of a Cell-Penetrating Peptide with Potential Anticancer Activity. *ChemMedChem* 2017, 12(1):42–49. doi: 10.1002/cmdc.201600498 27860402 PMC5516705

[26] Huang C, Liu F, Feng Q, Pan X, Yang JL: RGD4C Peptide Mediates anti-p21Ras scFv Entry Into Tumor Cells and Produces an Inhibitory Effect on the Human Colon Cancer Cell Line SW480. *BMC Cancer* 2020, 21(1):321. 10.1186/s12885-021-08056-4 PMC7993510 33765976

[27] Men A, Mrjb C, Mm A, D NS, Ma E, Hrs F, et al: The effect of RGD-targeted and non-targeted liposomal Galbanic acid on the therapeutic efficacy of pegylated liposomal Doxorubicin: From liposomal preparation to In-vivo studies—ScienceDirect. *International Journal of Pharmaceutics* 2021, 604(1):120710. 34019972 10.1016/j.ijpharm.2021.120710

[28] Zeng Z, Chen J, Luo S, Dong J, Hu H, Yang Z, et al: Targeting and imaging colorectal cancer by activatable cell-penetrating peptides. *Am J Transl Res* 2020, 12(5):1754–1766. 32509174 PMC7270030

[29] Yu D, Xinrui L, Qiang F, Xinyan P, Shuling S, Julun Y: Inhibition of human lung cancer cells by anti-p21Ras scFv mediated by the activatable cell-penetrating peptide *Anti-Cancer Drugs* 2021, 33(1):e562–e572. 10.1097/CAD.0000000000001180 PMC8670359 34338241

[30] Zhang X, Lin C, Lu A, Lin G, Chen H, Liu Q, et al: Liposomes equipped with cell penetrating peptide BR2 enhances chemotherapeutic effects of cantharidin against hepatocellular carcinoma. *Drug Deliv* 2017, 24(1):986–998. doi: 10.1080/10717544.2017.1340361 28644728 PMC8241055

[31] Shafiee F, Rabbani M, Jahanian-Najafabadi A: Optimization of the Expression of DT386-BR2 Fusion Protein in Escherichia coli using Response Surface Methodology. *Advanced Biomedical Research* 2017, 6(22):1–6. doi: 10.4103/2277-9175.201334 28349025 PMC5353773

