# Supplementary figures and images for: BR2 cell penetrating peptide effectively delivers anti-p21Ras scFv to tumor cells with ganglioside expression for therapy of ras-driven tumor
Source: PLoS One. 2022 Jun 1;17(6):e0269084. doi: 10.1371/journal.pone.0269084 (PMC9159597; doi:10.1371/journal.pone.0269084)

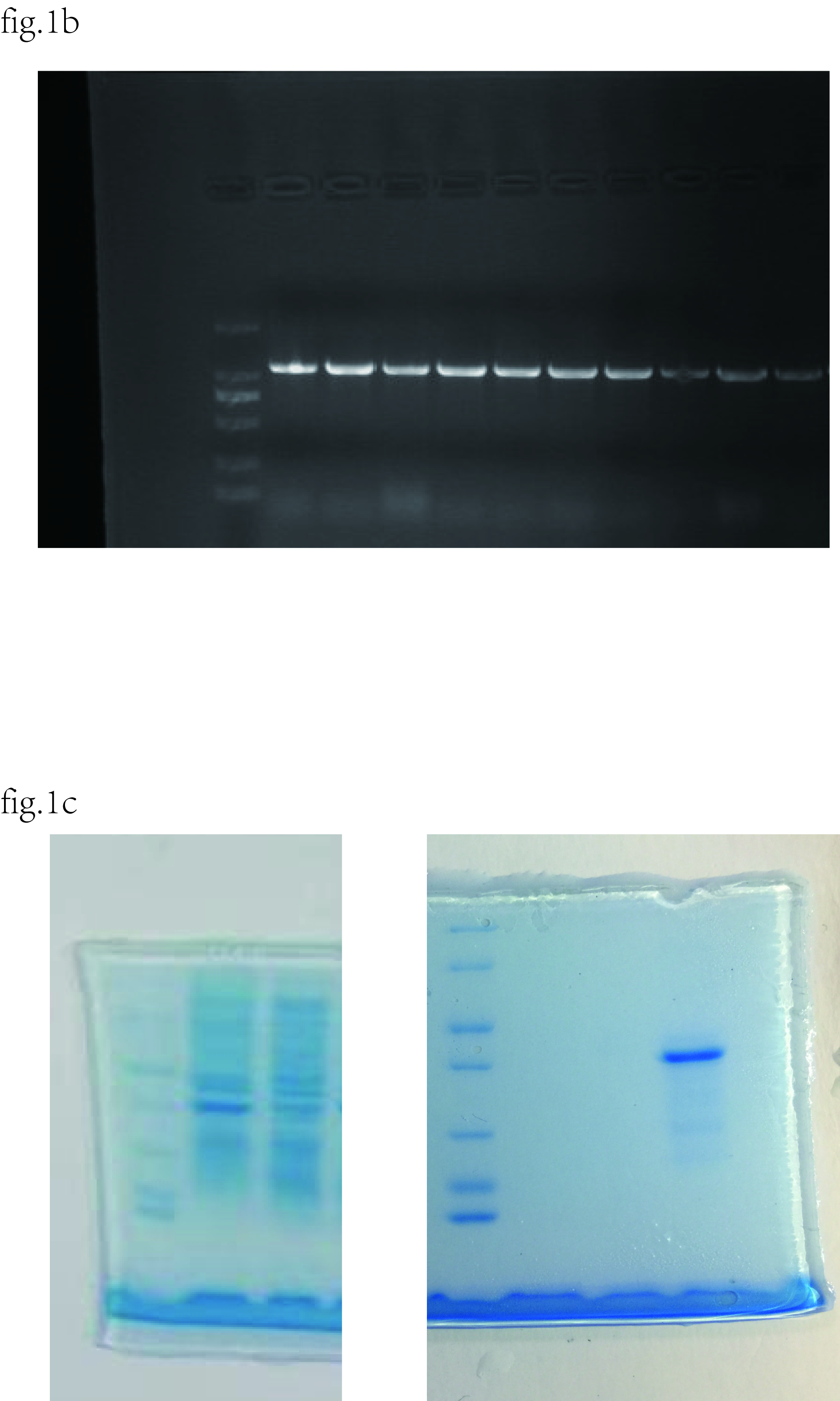

Supplement: S1 Fig — (TIF) [file pone.0269084.s001.tif]

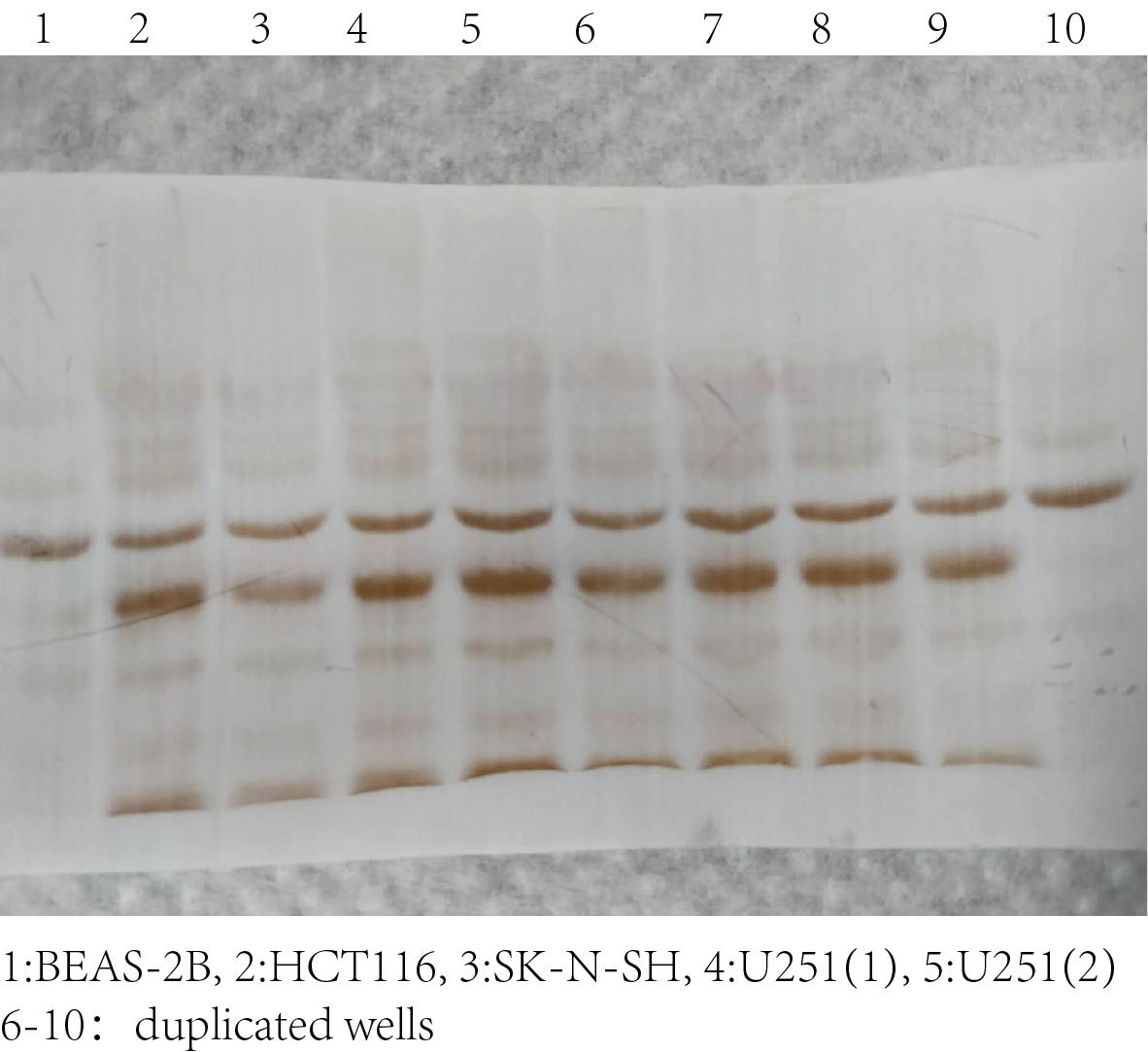

Supplement: S2 Fig — (PNG) [file pone.0269084.s002.png]
